# Peer Review of “Impact of the COVID-19 Pandemic on Routine Childhood Vaccination Coverage in Ecuador From 2019 to 2021: Comparative Analysis”

**DOI:** 10.2196/84848

**Published:** 2025-10-17

**Authors:** Adeleke Adekola

**Affiliations:** 1 Syracuse University, Syracuse, NY, United States

**Keywords:** COVID-19 pandemic, vaccination coverage, Ecuador, immunization, routine vaccination, health disparities, vaccine hesitancy

*This is the peer-review report for “Impact of the COVID-19 Pandemic on Routine Childhood Vaccination Coverage in Ecuador From 2019 to 2021: Comparative Analysis.”*

## Round 1 Review

### General Comments

This manuscript [1] presents a thorough and compelling analysis of the impact of the COVID-19 pandemic on routine and COVID-19 vaccination coverage in Ecuador. Using national and regional data from 2019 to 2021, the authors provide clear evidence of declining immunization rates across a range of vaccines, emphasizing the public health implications of disrupted vaccination services. The use of comparative and trend analyses, as well as spatial disaggregation by region, strengthens the study’s findings. The paper is timely, well-organized, and contributes valuable insights for health system resilience in the face of future public health crises.

### Specific Comments

#### Major Comments

1. Clarity on methodology: The study uses observational comparative analysis and descriptive statistics but would benefit from additional details on the specific statistical tests used (eg, Joinpoint regression) and any confidence intervals or measures of significance included.

2. Policy and programmatic implications: While the discussion clearly outlines the negative impact on vaccination coverage, the manuscript could be strengthened by offering more specific recommendations for public health policy, especially regarding catch-up campaigns or digital infrastructure improvements to track immunization.

3. Sociodemographic context: The analysis highlights disparities but could be improved by integrating more granular sociodemographic information (eg, income, ethnicity, rurality) to provide a deeper understanding of inequities in coverage and guide targeted interventions.

#### Minor Comments

4. Language and style: The manuscript would benefit from light editing to improve flow and reduce minor typographical and grammatical errors.

5. Figure/table integration: Tables are rich in data, but would be more useful if the text referenced key figures and included short interpretation notes to help readers navigate large data points.

6. Redundancy in the Introduction: Some repetition in the early paragraphs could be streamlined to maintain reader engagement.
